# Mutations in *SLC12A5* in epilepsy of infancy with migrating focal seizures

**DOI:** 10.1038/ncomms9038

**Published:** 2015-09-03

**Authors:** Tommy Stödberg, Amy McTague, Arnaud J. Ruiz, Hiromi Hirata, Juan Zhen, Philip Long, Irene Farabella, Esther Meyer, Atsuo Kawahara, Grace Vassallo, Stavros M. Stivaros, Magnus K. Bjursell, Henrik Stranneheim, Stephanie Tigerschiöld, Bengt Persson, Iftikhar Bangash, Krishna Das, Deborah Hughes, Nicole Lesko, Joakim Lundeberg, Rod C. Scott, Annapurna Poduri, Ingrid E. Scheffer, Holly Smith, Paul Gissen, Stephanie Schorge, Maarten E. A. Reith, Maya Topf, Dimitri M. Kullmann, Robert J. Harvey, Anna Wedell, Manju A. Kurian

**Affiliations:** 1 Department of Women's and Children's Health, Karolinska Institutet, SE-171 76 Stockholm, Sweden; 2 Neuropediatric Unit, Karolinska University Hospital, SE-171 76 Stockholm, Sweden; 3 Molecular Neurosciences, Developmental Neurosciences Programme, UCL Institute of Child Health, London WC1N 1EH, UK; 4 Department of Neurology, Great Ormond Street Hospital, London WC1N 3JH, UK; 5 Department of Pharmacology, UCL School of Pharmacy, London WC1N 1AX, UK; 6 Department of Chemistry and Biological Science, Graduate School of Science and Engineering, Aoyama Gakuin University, Sagamihara 252-5258 Kanagawa, Japan; 7 Center for Frontier Research, National Institute of Genetics, Yata 1111, Mishima, 411-8540 Shizuoka, Japan; 8 PREST, Japan Science and Technology Agency, Tokyo 102-0076, Japan; 9 Department of Psychiatry, New York University School of Medicine, New York, New York 10016, USA; 10 Institute of Structural and Molecular Biology, Crystallography/Department of Biological Sciences, Birkbeck College, University of London, WC1E 7HX, UK; 11 Laboratory for Developmental Biology, Graduate School of Medical Science, University of Yamanashi, Chuo, 409-3898, Japan; 12 Department of Neurology, Royal Manchester Children's Hospital, Manchester, M13 9WL, UK; 13 Academic Department of Radiology, Royal Manchester Children's Hospital, Manchester, M13 9WL, UK; 14 Imaging Science, School of Population Health, University of Manchester, Manchester, M13 9PL, UK; 15 Department of Molecular Medicine and Surgery, Science for Life Laboratory, Center for Molecular Medicine, Karolinska Institutet, SE-171 76 Stockholm, Sweden; 16 Centre for Inherited Metabolic Diseases, Karolinska University Hospital, SE-171 76 Stockholm, Sweden; 17 Department of Cell and Molecular Biology, Science for Life Laboratory, Uppsala University, SE-751 24 Uppsala, Sweden; 18 Department of Medical Biochemistry and Biophysics, Science for Life Laboratory, Karolinska Institutet, SE-171 21 Stockholm, Sweden; 19 EEG Department, Royal Oldham Hospital, OL1 2JH, Oldham, Lancashire, UK; 20 Young Epilepsy, RH7 6PW, Lingfield, Surrey, UK; 21 Department of Molecular Neuroscience, UCL Institute of Neurology, WC1N 3BG, London, UK; 22 Department of Laboratory Medicine, Karolinska Institutet, SE-171 76 Stockholm, Sweden; 23 Science for Life Laboratory, School of Biotechnology, Royal Institute of Technology, SE-100 44 Stockholm, Sweden; 24 Department of Neurological Sciences, University of Vermont College of Medicine, Vermont, VT 05405, USA; 25 Department of Paediatric Neurology, Fletcher Allen Health Care, Vermont, VT 05401, USA; 26 Clinical Neurosciences, Developmental Neurosciences Programme, UCL Institute of Child Health, London, WC1N 1EH, London, UK; 27 Department of Neurology, Epilepsy Genetics Programme, Boston Children's Hospital, Boston, Massachusetts, Massachusetts 02115, USA; 28 Department of Neurology, Harvard Medical School, Boston, Massachusetts, Massachusetts 02115, USA; 29 Department of Medicine and Paediatrics, University of Melbourne, Austin Health and Royal Children's Hospital, Melbourne, Victoria, VIC 3052, Australia; 30 Florey Institute, Melbourne, Victoria, VIC 3010, Australia; 31 MRC Laboratory for Molecular Cell Biology, UCL, London, WC1E 6BT, UK; 32 Department of Metabolic Medicine, Great Ormond Street Hospital, London, WC1N 3JH, UK; 33 Genetics and Genomic Medicine, Institute of Child Health, UCL, London, WC1N 1EH, UK; 34 Department of Clinical and Experimental Epilepsy, UCL Institute of Neurology, London, WC1N 3BG, UK; 35 Department of Biochemistry and Molecular Pharmacology, New York University School of Medicine, New York, New York 10016, USA

## Abstract

The potassium-chloride co-transporter KCC2, encoded by *SLC12A5*, plays a fundamental role in fast synaptic inhibition by maintaining a hyperpolarizing gradient for chloride ions. KCC2 dysfunction has been implicated in human epilepsy, but to date, no monogenic KCC2-related epilepsy disorders have been described. Here we show recessive loss-of-function *SLC12A5* mutations in patients with a severe infantile-onset pharmacoresistant epilepsy syndrome, epilepsy of infancy with migrating focal seizures (EIMFS). Decreased KCC2 surface expression, reduced protein glycosylation and impaired chloride extrusion contribute to loss of KCC2 activity, thereby impairing normal synaptic inhibition and promoting neuronal excitability in this early-onset epileptic encephalopathy.

## Abstract (web summary)

The potassium-chloride co-transporter, KCC2 is an essential component in maintaining a gradient for chloride ions in neurons. Here Stodberg and colleagues identify loss-of-function mutations in the encoding gene *SLC12A5*, which impair normal synaptic function associated with early-onset epilepsy.

## Graphical abstract

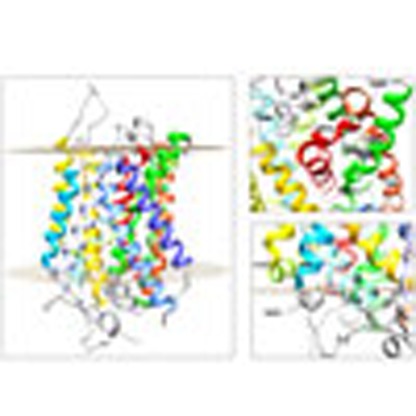

Epilepsy of infancy with migrating focal seizures (EIMFS) is an early-infantile epileptic encephalopathy (EIEE) previously known as migrating partial seizures of infancy. It is characterized by multifocal seizures, developmental arrest or regression and a distinct ictal pattern on electroencephalogram (EEG)^1^,^2^. EIMFS is genetically heterogeneous^1^,^2^,^3^ (Supplementary Table 1) and the underlying cause remains elusive for many affected infants^1^.

In this study, we report a novel genetic cause of EIMFS, identifying recessive *SLC12A5* mutations in four affected children from two unrelated families. *SLC12A5* encodes the potassium-chloride co-transporter KCC2, which plays an integral role in neuronal inhibition^4^,^5^,^6^,^7^ and maturation of cortical dendritic spines^8^. We demonstrate that the identified mutants negatively impact KCC2 protein expression and glycosylation, with impaired KCC2-mediated chloride extrusion.

## Results

### Recessive *SLC12A5* mutations in patients with EIMFS

**Figure 1:**
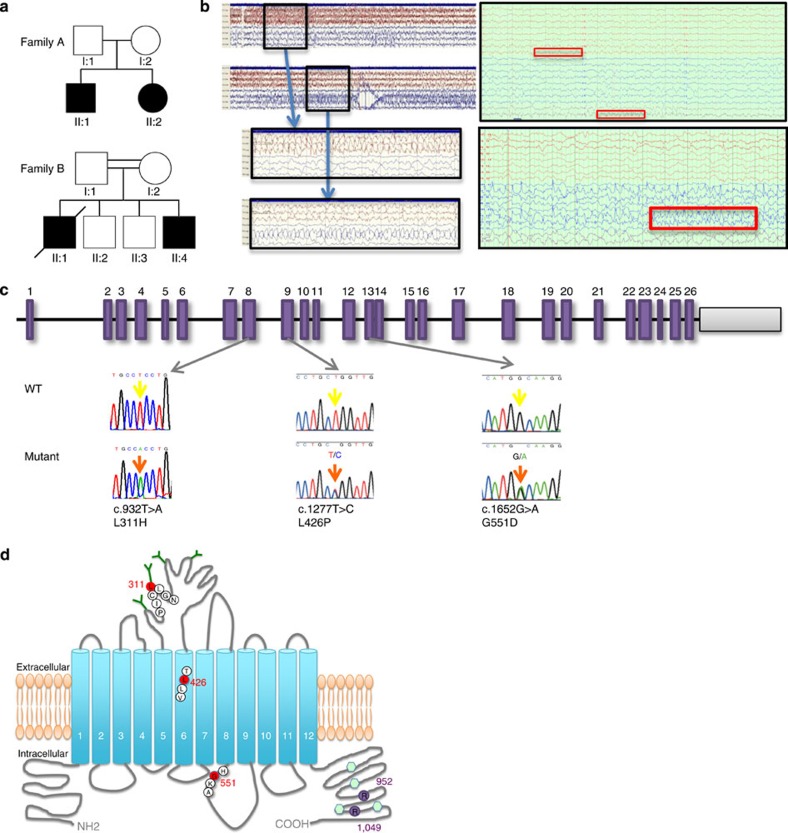
Clinical and molecular genetic findings in families with EIMFS. (**a**) Family A (Caucasian origin) and Family B (Pakistani origin). Squares represent males. Circles represent females. Affected individuals are represented by black shading. Double parallel horizontal bars indicate consanguinity in Family B. (**b**) The left upper and lower EEG recording are two consecutive EEG segments, each 1 min long, from the ictal EEG of proband A-II:1. Seizure activity is boxed in black with rhythmic spike-waves evident initially on the left hemisphere (red), then fading and seizure activity starting up in the right (blue) temporal area. Expanded segments below (10 s) show the spike-wave activity in more detail. Montage: Longitudinal bipolar according to the 10–20 system. Only lateral channels shown. The right upper (compressed) and lower EEG recording from proband B-II:4 reveal central/vertex and right parietal epileptiform activity (purple/red and boxed in red, upper right figure) that wanes with a return to the interictal state before onset of left centro-temporal ictal activity associated with facial twitching (blue and boxed in red, lower right figure). (**c**) *SLC12A5* consists of 26 exons (purple rectangles). Both wild-type (WT) and mutant DNA sequence is displayed. Three point mutations were identified in four patients with EIMFS (WT sequence indicated by yellow arrow, mutated base by orange arrow). The children from Family A harboured missense mutations in exon 9 (c.1277T>C, L426P) and 13 [c.1652G>A, G551D], while the two affected Family B patients were homozygous for missense variant c.932T>A (L311H) in exon 8. (**d**) The KCC2 protein structure consists of 12 transmembrane (TM) helices (numbered blue cylinders) interconnected by a series of extracellular and intracellular loops. Sites of phosphorylation at the C-terminus are depicted as light green hexagons. N-linked glycosylated sites are indicated as dark green Y-shaped structures present at the extracellular loop between TM5 and TM6. The EIMFS mutations are indicated in red circles, located in the extracellular loop between TM5 and TM6 (L311H), within TM6 (L426P) and in the intracellular loop between TM8 and TM9 (G551D). Variants associated with idiopathic generalized epilepsy and febrile convulsions are represented as purple circles at the C-terminus.

From a cohort of 42 patients (39 families) with EIMFS, we identified two families, one kindred of Swedish origin (Family A, unrelated asymptomatic parents) and one of Pakistani descent (Family B, consanguineous first cousin healthy parents). Each family had two affected children who developed clinical features of EIMFS (Fig. 1a). All four patients presented similarly with infantile-onset focal migrating seizures, with rapid escalation to a severe pharmacoresistant epileptic disorder (Supplementary Note 1). Interictal multifocal epileptiform discharges were observed on EEG, and ictal activity migrating from one hemisphere to the other was recorded (Fig. 1b). In all cases, there was significant neurological regression leading to severe developmental delay. One patient died (at the age of 2.5 years) due to secondary cardiorespiratory complications (Supplementary Note 1, Supplementary Fig. 1).

Using an autozygosity mapping strategy in kindred B, and exome sequencing in both Families A and B, we identified monogenic recessive *SLC12A5* mutations in all affected children with EIMFS. Exome sequencing for both parents and children of Family A was undertaken and all variants scored and ranked using the Mutation Identification Pipeline weighted sum model (Supplementary Table 2) that uses multiple parameters, but emphasizes Mendelian inheritance patterns, conserved, rare and protein damaging variants. This left only one gene, *SLC12A5*, with adequate coverage in all individuals sequenced that conformed to autosomal recessive inheritance (Supplementary Table 3a). For Family B, single-nucleotide polymorphism (SNP) array analysis revealed seven common regions of homozygosity (6–43 Mb in size) in the affected children (Supplementary Table 3b). Stringent filtering for homozygous, potentially pathogenic variants within these homozygous regions identified four genes, of which, after detailed analysis (Supplementary Methods, Supplementary Table 3b) *SLC12A5* was deemed by far the most biologically plausible candidate for this phenotype. Results from exome analysis were confirmed on direct Sanger sequencing. The children from Family A were confirmed compound heterozygotes for missense mutations c.1277T>C (L426P) and c.1652G>A (G551D), while the two affected patients from Family B were homozygous for the missense variant c.932T>A (L311H) (Fig. 1c,d). All mutations showed appropriate familial disease segregation (parents were heterozygous carriers) and were not reported in healthy control populations (dbSNP137, 1000 Genomes SNP calls and the NHLBI Exome Sequencing Project). Affected amino acids were highly conserved across species (Supplementary Fig. 2). In both families, no potentially pathogenic variants were found in other genes known to cause EIMFS or EIEE. A further 38 patients (from 37 families) with EIMFS were screened for mutations in *SLC12A5* and no mutations were identified (Supplementary Methods). There were no discernible phenotypic differences between the *SLC12A5* mutation-positive and mutation-negative patients.

### KCC2 mediates physiological neuronal chloride extrusion

KCC2 is a membrane protein located at the somatodendritic cell compartment of mature neurons^4^. KCC2 is exclusively expressed in the central nervous system, including neurons in all layers of the neocortex, CA1-4 subfields of the hippocampus and cerebellum^4^,^5^. In mature neurons, KCC2 is the major extruder of intracellular chloride and in the presence of low intraneuronal chloride levels, the binding of GABA (γ-aminobutyric acid) and glycine to their ionotropic receptors results in chloride influx with subsequent hyperpolarization contributing to neuronal inhibition^4^,^5^. During brain development, the neuronal chloride equilibrium potential (E_CI_) gradually hyperpolarizes, in parallel with upregulation of KCC2, which contributes to the establishment and maintenance of hyperpolarizing inhibitory post-synaptic potentials^6^,^7^. Recent evidence additionally implicates KCC2 as a structural protein involved in the morphological and functional maturation of cortical dendritic spines^8^. A number of further investigations were therefore undertaken to elucidate the impact of the identified mutations on KCC2 function.

### Homology modelling for identified KCC2 mutants

**Figure 2:**
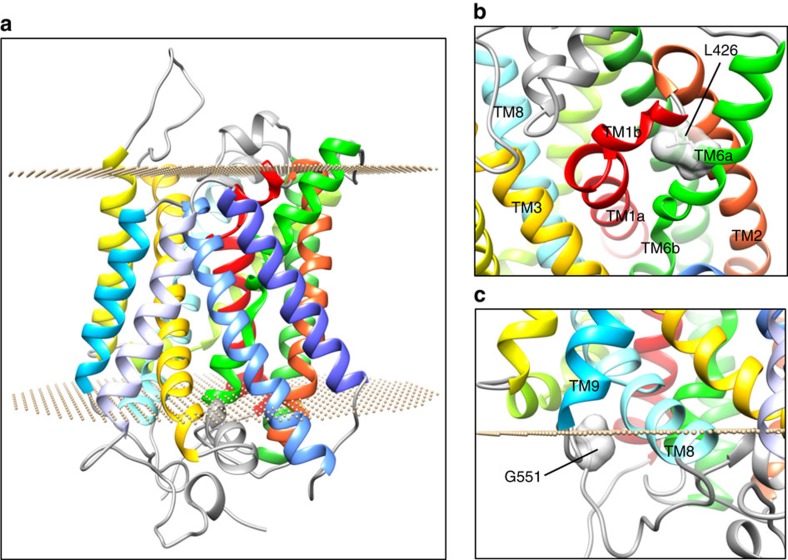
Structural modeling of KCC2 mutants. (**a**) A homology model of KCC2 showing a transmembrane (TM) protein consisting of 12 TM helices interconnected by intracellular and extracellular loops. (**b**) In our homology model, L426 (shown in a surface representation) is located in the unwound TM6 (TM6a). This helix, together with TM1, is pivotal for the transporter function. In the L426P mutant, the substitution of a leucine with a proline (not shown) is likely to introduce a kink in TM6, which will result in alternation of the interaction with TM1b. A comparative analysis of different structures of transporters (LeuT, vSGLT and ApcT structures) shows that the conformation of TM1b plays a key role in allowing access to the substrate binding pocket (maintaining a conformation that completely occludes access to the post-synaptic space but only partially occludes access to the cytosolic side). Thus, the presence of a proline in KCC2 TM6 could lead to an alteration of the extracellular occlusion formed by TM1b and TM6. (**c**) Our KCC2 model predicts that G551 (shown in a surface representation) is located in the intracellular loop between TM8 and TM9 (residues 539–557). In the G551D mutant, the substitution of the glycine with an aspartic acid—a larger and negatively charged amino acid—is likely to introduce a distortion in the orientation of TM8. The latter helix contains the putative substrate binding residues A521 and S525, based on homology to ApcT (A287 and T291, respectively, PDB: 3GIA)^35^. Therefore, introducing an aspartic acid in position 551 could lead to disruption of the binding site thereby affecting substrate binding.

KCC2 exists in both monomeric and oligomeric forms^9^, and consists of 12 transmembrane (TM) helices interconnected by a series of extracellular and intracellular loops (Figs 1d,2a). Alternative splicing gives rise to two isoforms KCC2a and KCC2b, differing by an extra 40 amino acids in the N-terminus of KCC2b. The changes identified in our patients are present in both isoforms, and protein homology modelling studies were undertaken to predict damaging effects on the structure-function properties of KCC2 (Fig. 2a-c). Located within TM6, L426 is almost universally conserved in the 100 most similar proteins and substitution with a proline residue is expected to disrupt this TM helix (Fig. 2b). G551, located in the intracellular loop between TM8–TM9 (Fig. 2c), is also well conserved in similar proteins, and substitution with the significantly larger negatively charged aspartate residue may interact unfavourably with other residues in the loop region or block other molecular interactions. L311H is located extracellularly between TM5 and TM6 and the extended loop region between residues 303 and 407 is not amenable to accurate modelling because of lack of sequence identity. Most KCC2 homologues have a hydrophobic residue at this position 311 (typically valine, methionine, isoleucine or phenylalanine), and a leucine/histidine exchange would therefore be expected to break hydrophobic interactions, thereby disrupting structural stability of the TM5–TM6 loop. L311 is also adjacent to one of four evolutionarily conserved cysteines (C310, C325, C345 and C354) in the TM5–TM6 region that are vital for KCC2 transport activity^10^. L311 may be part of a hydrophobic pocket formed with a cysteine disulphide bond, in which case L311H may also interfere with the formation of intra- or intermolecular disulphide bonds.

### KCC2 mutants affect chloride homeostasis

**Figure 3:**
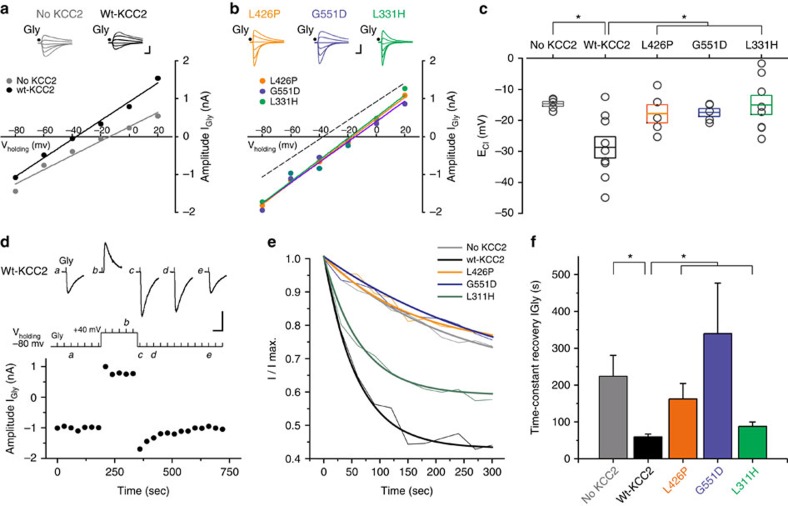
Electrophysiology of wild-type and mutant KCC2. (**a**–**c**) KCC2 mutants exhibit a depolarized E_Cl_. (**a**) Examples of I–V relationships in HEK293 cells transfected either with GlyR α_2_ alone (no KCC2), or GlyR α_2_ with a wild-type KCC2 construct. I_Gly_ amplitude was plotted against the holding potential and the intercept of the line of fit of this relation with the abscissa was taken as E_Cl_. Superimposed traces show examples of glycine-evoked currents recorded from individual cells at different holding potentials (–80 to +20 mV). (**b**) Examples of I–V relationships in HEK293 cells co-transfected with GlyR α_2_ and mutant KCC2, as indicated. E_Cl_ in mutant KCC2 is depolarized in comparison to wild-type (fit of I–V relation indicated by dashed-line). Calibration bars for 3a and 3b: 1nA, 1sec. (**c**) Summary chart plotting the distribution of E_Cl_ for each cell group: no KCC2: –14.7±0.8 mV (*n*=5), GlyR-KCC2: –29±3.5 mV (*n*=9), G551D: –17.4±1.2 mV (*n*=5), L426P: –17.9±2.9 mV (*n*=5), L311H: –15±3.1 mV (*n*=9); *P=0.006, one-way analysis of variance (ANOVA). Grey symbols represent measurements from individual cells. Boxes represent the mean±standard error of the mean. GlyR α_2_: glycine receptor α_2_, Wt-KCC2: wild-type K^+^-Cl^−^ co-transporter. (**d**–**f**) The rate of Cl^−^ extrusion is impaired in KCC2 mutants. (**d**) I_Gly_ amplitude plotted against time in a HEK293 cell co-transfected with GlyRα_2_ and wild-type KCC2 and held at different membrane potentials as indicated in the top image. Vertical bars indicate the times of successive glycine applications. Example traces show glycine-evoked currents at times indicated by letters in italic. Calibration bars: 1 nA, 300 ms. (**e**) Rate of recovery of I_Gly_ amplitude after switching back the holding potential from +40 to −80 mV. Fine lines replace symbols and error bars are omitted for legibility. An exponential decay function was used to fit I_Gly_ amplitude ratio in each cell group. (**f**) Summary bar graph of the time-constant of I_Gly_ recovery in each group (no KCC2: 224±56.7 s, *n*=6; WT-KCC2: 59.5±7.7 s, *n*=13; L426P: 162.5±42.1 s, *n*=4; G551D: 340.1±137 s, *n*=4; L311H: 88.1±11.5 s, *n*=7). *Different from WT-KCC2 (*P*<0.01), ANOVA.

To investigate the functional consequences of the identified mutations on KCC2 transporter function and chloride (Cl^−^) homeostasis, we recorded in voltage-clamp mode from HEK293 cells transiently transfected with enhanced green fluorescent protein (eGFP) and the glycine receptor (GlyR) α_2_ subunit, which forms homopentameric glycine-gated chloride channels^11^. Recordings were also obtained from cells co-transfected with a plasmid either encoding the wild-type KCC2b isoform or one of the mutants L311H, L426P and G551D. Pressure application of glycine evoked large inward currents (I_Gly_) in eGFP-positive cells held at –80 mV (*n*=33), but not in eGFP-negative cells (*n*=8). The I_Gly_ amplitude varied linearly with the holding potential and reversed in polarity. Fitting the I–V relationship, cells transfected with both GlyR α_2_ and wild-type KCC2 yielded a more hyperpolarising E_Cl_ than cells solely expressing GlyR α_2_ (Fig. 3a), consistent with Cl^−^ extrusion by KCC2. KCC2 mutants, however, exhibited a depolarized E_Cl_ relative to wild-type KCC2 (Fig. 3b). The estimated E_Cl_ for all three mutants was similar and did not differ significantly from that in cells solely expressing GlyR α_2_ and eGFP (Fig. 3c), consistent with passive equilibration of Cl^−^ ions as predicted from the pipette and extracellular solutions. To further explore impaired mutant KCC2 activity, we measured the rate at which E_Cl_ recovers to a starting value after a period of chloride load, as described previously^12^. Glycine was applied every 30 s while holding cells at −80 mV then at +40 mV to load them with Cl^−^ (Fig. 3d). When the holding potential was reset to −80 mV, glycine-evoked currents were larger due to the increased driving force. In cells transduced with wild-type KCC2, glycine-evoked currents I_Gly_ returned to the initial amplitude consistent with transporter-mediated extrusion of chloride. The rate at which I_Gly_ amplitude recovered for the three KCC2 mutants was much slower (Fig. 3e,f). The recovery rate in cells expressing L426P and G551D mutants was not significantly different from cells expressing only GlyR α_2_ and eGFP, consistent with near complete loss of KCC2 function. The recovery rate for the L311H mutant was intermediate, suggesting some residual KCC2 activity. Thus, the identified missense changes in KCC2 render the transporter either near non-functional (L426P and G551D) or partly functional (L311H), and are predicted to lead to intracellular Cl^−^ accumulation in neurons with loss of a hyperpolarizing driving force for GABA_A_Rs and GlyRs.

### Mutant KCC2 shows reduced expression/altered glycosylation

**Figure 4:**
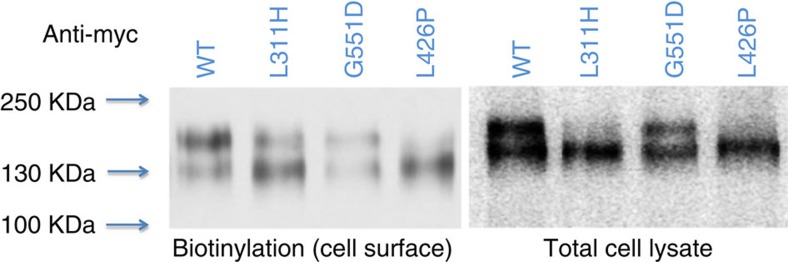
Immunoblotting studies for KCC2 mutants. Immunoblotting studies including cell surface biotinylation and total cell lysate studies. Two bands are observed for WT and the three mutant KCC2 proteins closely located to each other at ∼130 kDa and corresponding to glycosylated (upper band) and unglycosylated (lower band) states^36^.

**Figure 5:**
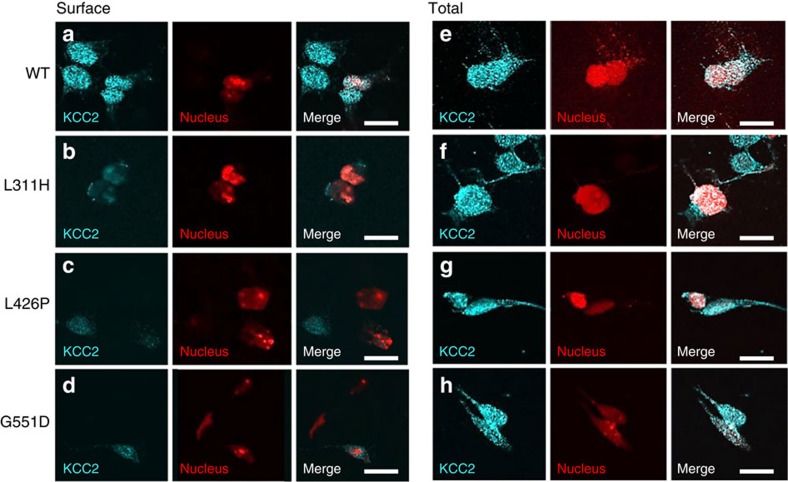
Immunofluorescence microscopy for KCC2 mutants. L311H, L426P and G551D substitutions impair cell-surface expression of KCC2. HEK293 cells were co-transfected with KCC2-FLAG and HsRed-NLS and immunostained using anti-FLAG and AlexaFluor 488 antibodies. KCC2 WT is detected at the cell surface of intact cells (**a**) whereas KCC2-L311H, -L426P and -G551D display very little cell surface expression (**b**–**d**) Expression of KCC2-WT,-L311H,-L426P and -G551D is detected in permeabilised cells (**e**–**h**) Scale bar is 20 μm.

To further investigate disease mechanisms in KCC2-associated EIMFS, an *in vitro* heterologous expression system for immunoblotting and surface protein biotinylation was used to compare total and cell surface expression levels of wild-type and mutant KCC2 (Fig. 4). When compared with wild-type, the mutants L311H, L426P and G551D showed significantly reduced KCC2 expression at the cell surface versus total (whole-cell lysate) expressed transporter (two bands representing glycosylated and unglycosylated protein occur at ∼130 kDa, Fig. 4). The proportion of wild-type KCC2 existing in the glycosylated state was 50% at the surface and 43% in whole-cell lysate; significantly lower proportions were observed for all three mutants at both the cell surface and in total cell lysates (Table 1). The missense changes therefore appear to impair cell surface expression and post-translational modification of KCC2. To further investigate the cell surface expression of KCC2 mutants, HEK 293 cells were transfected with KCC2, FLAG-tagged at the second extracellular loop, and HcRed NLS, to visualize the cell nucleus and provide confirmation of effective transfection. Immunostaining of permeabilised cells revealed that all three mutants were overall expressed at similar levels to wild-type KCC2 (Fig. 5e–h). However, while wild-type KCC2 was robustly detected at the surface of intact cells, much less KCC2 was detected in cells expressing KCC2-L311H, -L426P and -G551D (Fig. 5a–d), further suggesting that these three mutations lead to expression of mutant KCC2 transporters with impaired cell-surface localization.

### KCC2 knockout zebrafish model reveals early motor deficit

A zebrafish disease model was utilized to study the effect of KCC2 deficiency on circuit function at an early stage of development. Zebrafish have two KCC2 orthologs, KCC2a and KCC2b (due to ancestral gene duplication) and TALEN-mediated genome editing was undertaken to generate knockout zebrafish models. An 8-bp deletion was induced in exon 4 of *KCC2a* to disrupt KCC2a and similarly, a 5-bp deletion was induced in exon 4 of *KCC2b* to disrupt KCC2b. Wild-type zebrafish larvae at 2 days post fertilization (dpf), showed a typical escape swimming response upon touch, with rhythmic side-to-side contraction of the trunk (Supplementary Video 1). Neither single KCC2a nor KCC2b knockout zebrafish displayed developmental or motor defects, and both survived into adulthood. A KCC2a-KCC2b double knockout zebrafish model was then generated, and tactile-induced motor behaviour was assessed. In contrast to wild-type, the KCC2a-KCC2b double knockout zebrafish showed abnormal jerky spasmodic movements during the escape response (Supplementary Video 2). Severe motor phenotypes are also reported in other loss-of-function KCC2 animal models. Null mutations in *Kazachoc* (*Drosophila* ortholog of *KCC2*) lead to lethality, while partial loss-of-function causes increased seizure susceptibility mediated by GABA_A_ receptor dysfunction.^13^ Complete knockout of *kcc2* in mice also leads to neonatal lethality^14^. A murine model retaining ∼5% KCC2 function displays frequent spontaneous seizures and excess hippocampal excitability^15^. Mice with one null and one hypomorphic allele resulting in 15–20% overall residual KCC2 protein have an increased susceptibility to induced seizures with abnormal cognitive function^16^. Overall, our data suggests that, as evident in a number of other animal models, loss of KCC2 function causes an early motor deficit in zebrafish, characterized by jerky spasmodic movements. While the double knockout zebrafish may represent a useful model for EIMFS, true comparability to the human disease is limited by genetic and biological differences between the two model systems.

### KCC2 in epilepsy and other neurological disorders

**Figure 6:**
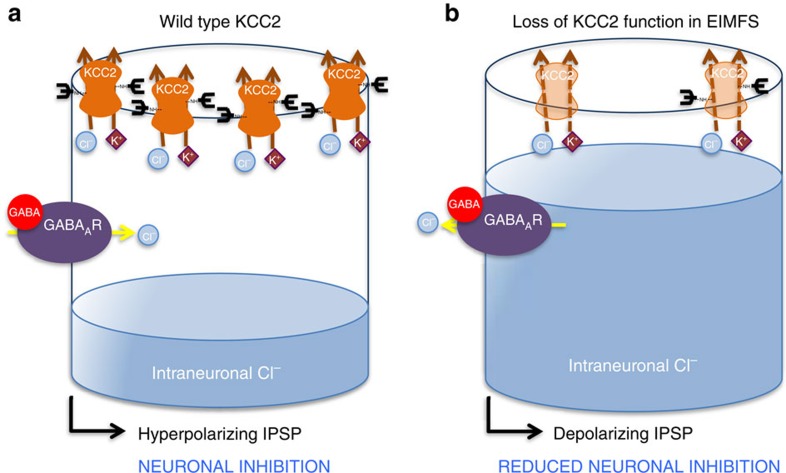
Schematic representation of postulated disease mechanisms in KCC2-EIMFS. A post-synaptic neuron is schematically represented for (**a**) wild-type and (**b**) mutant KCC2 in EIMFS. Neurotransmitters and ions are depicted as shapes, namely GABA (red circle), potassium (K^+^, magenta diamond) and chloride (Cl^−^, blue circle). The mature KCC2 transporter (orange) with N-linked glycosylated extracellular domains (black branched structure) is located at the synaptic membrane. The GABA_A_ receptor (GABA_A_R) is a purple oval. In normal mature neuronal cells (**a**) KCC2 transporter (bold orange) maintains low intraneuronal chloride (represented by blue fluid level) through chloride extrusion (solid orange lines). With GABA binding, the resultant gradient allows an influx of chloride via the GABA_A_R resulting in a hyperpolarizing IPSP (inhibitory post-synaptic potential), which contributes to neuronal inhibition. In KCC2-EIMFS (**b**) a number of mechanisms contribute to loss of KCC2 function (faded orange KCC2 transporter) and impaired transporter ability to extrude chloride (dashed orange lines), including reduced cell surface transporter expression and abnormal protein glycosylation. This results in a depolarizing inhibitory postsynaptic potential, thereby leading to impaired neuronal inhibition.

The physiological role of KCC2 in mediating neuronal inhibition has led to considerable speculation about the potential involvement of this transporter in the pathogenesis of human epilepsy^17^. Our report of autosomal recessive loss-of-function *SLC12A5* mutations in EIMFS substantiates this hypothesis. A role for KCC2 in human epilepsy is further evident by the recent identification of heterozygous missense *SLC12A5* polymorphisms in other epilepsy phenotypes, as R952H and R1049C exhibit statistical association with idiopathic generalized epilepsy^18^. These variants reside in conserved residues in the KCC2 cytoplasmic C-terminus and display reduced Cl(^-^)-extrusion capacities leading to less hyperpolarized glycine equilibrium potentials (E_Gly_), and impair KCC2 stimulatory phosphorylation at serine 940, a key regulatory site. Furthermore, R952H has been reported in an Australian family with febrile seizures^19^, and KCC2-R952H reduces neuronal Cl^−^ extrusion with impaired ability to induce dendritic spines. Reduced surface expression of KCC2-R952H is postulated to contribute to these functional deficits^19^. Pathogenic processes governing KCC2-related seizure disorders therefore appear multifactorial, with mechanistic overlap between the different epilepsies. Impaired KCC2-mediated chloride extrusion (Fig. 6) from defective transporter activity, decreased cell-surface KCC2 expression and reduced protein glycosylation contribute to loss of KCC2 function in the severe early-onset epileptic encephalopathy described in this report. Developing novel therapeutic molecules that increase the expression and efficacy of KCC2 for early clinical translation are now a research priority. Genotype-phenotype correlations will become evident over time, as more cases are reported, but *SLC12A5* gene dosage (recessive versus dominant disorders), mutation type and the effect of mutations on protein activity may indeed influence epilepsy phenotype, including age of disease onset and clinical severity. Identification of additional *SLC12A5* mutations in humans will no doubt further expand the clinical spectrum and solidify genotype-phenotype observations in KCC2-related human diseases.

## Methods

### Subject recruitment

Family A was enrolled in a Swedish study investigating EIEE with exome sequencing at the Karolinska University Hospital. Family B was recruited to a United Kingdom EIMFS genetic study from Royal Manchester Children's Hospital. In addition, patients with EIMFS (without mutations in known disease genes) were identified through international collaborators. This included 18 UK patients, 1 Swedish patient, 7 patients from the USA and 12 patients from an Australian cohort. Detailed electroclinical phenotyping was performed in all cases. Written informed consent was obtained from participants, and all studies were approved by local ethics committees (The Regional Ethical Review Board in Stockholm, Sweden and South Birmingham Research Ethics Committee, UK) and performed in accordance with the Declaration of Helsinki. Whole exome sequencing data were anonymised and deposited in secure databases at each participating academic institution with data access to members of the research teams involved in each study. Consent given by participants did not extend to public access of the sequence data given the risk of patient confidentiality issues.

### SNP array studies

Family B comprised two affected brothers born to first cousin parents so homozygosity mapping was undertaken. SNP genotyping was performed using Illumina cytoSNP-12 (Illumina Inc., San Diego, CA, USA). Both parents, two affected children and the two unaffected children were analysed. Genotypes were generated using GenomeStudio (Illumina Inc.) and then analysed manually using Microsoft Excel and also using BEDTools^20^.

### Exome sequencing

For exome sequencing, 2–5 μg of genomic DNA from Patients A-II:1, A-II:2, and both parents and B-II:4 was used to prepare paired-end libraries using TruSeq chemistry according to standard protocols (Illumina Inc.). Exome enriched libraries were made using the in-solution Nimblegen SeqCap EZ Exome Library (Roche Nimblegen Inc., Madison, WI, USA), Agilent SureSelect version 5 (Agilent) or standard protocols supplied by Illumina. Exome enriched DNA libraries were sequenced, 2 × 101 bases on a HiSeq 1000, 2000 or 2500 sequencing system (Illumina Inc.).

In Family A, bioinformatic analysis was performed using the Mutation Identification Pipeline (MIP) v.1.5.6 http://mip-api.readthedocs.org/en/latest/index.html. The data were annotated, scored and ranked using MIP. MIP annotates the variants with information from external and in-house databases as well as the genetic inheritance pattern (including compound mutations; Supplementary Table 2). A weighted sum model based on a subset of these annotations is then applied to generate a rank score for each variant. The variants are then sorted according to disease causing potential based on the rank score. Most weight in the rank score model is put on the inheritance pattern, minor allele frequency, gene coding annotation, functional annotation and protein predictions. Thus, MIP retains all variants but prioritizes them according to disease causing potential based on the rank score. Briefly, the sequence reads were aligned to the whole human genome reference GRCh37 using Mosaik (v.2.2.3) and PCR and optical duplicates were marked using PicardTools (Picard 2013, http://picard.sourceforge.net:). GATK package^21^,^22^ v.2.8-1 performed realignment, base recalibration, variant identification, recalibration and genotyping. Annovar^23^ v2013-08-23 annotated the variants and MIPs custom scripts ranked them according to pathogenic potential. All variants were loaded into Scout v.1.0 hosted at Science for Life Laboratory, Stockholm, for clinical evaluation.

In Family B the sequencing data were mapped to the canonical chromosomes (chr1-22, X, Y and M; UCSC genome browser) of the hg19 (GRCh37) reference human genome using the Novoalign Software. After removal of PCR duplicates (MosaikDupSnoop/Picard) and reads without a unique mapping location (MosaikSort), quality score recalibration and indel realignment were performed using the GATK package^21^,^22^. Variants were extracted using the Maq model in SAMtools^24^ and filtered by the following criteria: coverage >8 × , consensus quality >30, SNP quality >30 and root mean square mapping quality >30. These variants were further filtered against dbSNP build 135 and 1000 Genomes and annotated based on the UCSC known Canonical transcript database (UCSC genome browser) using Annovar. Genes containing non-synonymous or splice-site variants or coding indels were considered further. Due to the family structures for both families, we employed an autosomal recessive model, and genes containing one or more homozygous variants (Family A and B) or two or more heterozygous non-synonymous, splice-site or coding indels variants (Family A) were considered candidate genes. The candidate genes left after filtering were investigated for putative involvement in EIMFS by literature and database searches.

### Direct sanger sequencing

Sanger sequencing was used to confirm the variants identified on exome sequencing and establish segregation within each family. Primer pairs were designed for all 26 coding exons and exon/intron borders of *SLC12A5* for both transcripts ENST00000243964 and ENST00000454036 (www.ensembl.org). Experimental conditions (including primer sequences) are available on request. Sequencing reactions were run on an ABI PRISM 3730 or 130xl DNA Analyzer (Applied Biosystems Inc.).

### Molecular genetic screening of additional patients

EIMFS patients (total *n*=38) were also screened for *SLC12A5* variants, either by direct sequencing (*n*=14) or by analysis of pre-existing exome sequencing data (*n*=24). *SLC12A5* was screened in additional patients with EIEE who did not fit the electroclinical criteria of EIMFS (*n*=11).

### Homology modelling of KCC2

Fold recognition of KCC2 was performed with HHPRED^25^. The crystal structure of the *Methanocaldococcus jannaschii* ApcT Transporter (PDB: 3GIA; 2.35 Å resolution) was identified as the best template for residues 115–682 (TM middle domain), (HHPred e-value: 2.4 × 10^−36^). The HHpred alignment was used as preliminary pairwise sequence alignment for homology modelling. The extended loop region between TM5 and TM6 (residues 303–407) could not be accurately modelled due to lack of sequence identity and was therefore removed. A preliminary homology model of KCC2 middle domain was calculated using MODELLER-9v10^26^. The ConSurf web server^27^ with default parameters, was used to evaluate the evolutionary conservation and improve the quality of the alignment accordingly. This alignment was then used to create 1,000 homology models that were further assessed with the DOPE statistical potential score^28^. The model based on the lowest score (Normalized DOPE z-score: −0.464) was selected. In addition, evaluation using the ProSA web server showed that the model quality (*Z*-score=−2.63) fell within a range typically found for native proteins of similar size^29^. Next, selected non-synonymous substitutions were modelled into the KCC2 homology model with the swapaa command in Chimera^30^ using the Dunbrack backbone-dependent rotamer library^31^ and taking into account the lowest clash score, highest number of H-bonds and highest rotamer probability.

### Cell culture and transfection for electrophysiology

An expression vector (pCMV6-XL5) containing the full-length human *SLC12A5* cDNA was obtained from Origene, USA and validated by Sanger DNA sequencing. Cell-based cloning into a pRK5 expression vector (courtesy of Professor R. Harvey) using custom-designed cloning primers (Supplementary Table 4a) was successful for the wild-type DNA and for one of the mutations, For the remaining two mutations, custom-designed mutagenesis primers (Supplementary Table 4b) and a QuikChange II site-directed mutagenesis kit (Agilent Technologies, USA) were utilized. Plasmids containing each of the three mutations were thus generated^32^. Sanger sequencing was performed to ensure only the desired mutations had been introduced. HEK293 (ATCC CRL-1573, ATCC, Middlesex, UK) cells were transiently co-transfected (Lipofectamine) with plasmids encoding GFP, the human GlyR α2 subunit and equal amounts of WT, L311H, L426P or G551D KCC2.

### Electrophysiology

Patch pipettes (3–5 MΩ) were pulled from borosilicate glass (1.5-mm outer diameter, 0.5-mm wall thickness) and recordings were obtained from eGFP-positive HEK293 cells under infrared differential interference contrast imaging. The perfusion solution contained (in mM): NaCl (140), KCl (4), MgCl_2_ (1), CaCl_2_ (2), D-glucose (5), HEPES (10) (pH 7.2, osmolarity 298 mOsm.l^–1^). Currents were recorded with a Multiclamp 700-B amplifier (Molecular devices), filtered at 2 kHz (internal four-pole low-pass Bessel filter) and sampled at 10 kHz. The access resistance, monitored throughout the experiments was <15 MΩ and results were discarded if it changed by more than 20%. The pipette solution contained (in mM): CsCl (50), NaCl (10), CsF (60), EGTA (20), HEPES (10) (pH 7.2, osmolarity 310 mOsm.l^–1^). Local pressure application of glycine (1 mM in control HEPES solution) was delivered via a patch pipette connected to a Picospritzer (General Valve Corporation). The puff pipette was positioned roughly 10–20 μm away from the recorded cell and the bath perfusion arranged to keep it downstream of this position. Cells were first held at –80 mV while several glycine applications were made at 30 s intervals to ensure that no desensitisation occurred. Glycine puffs (3–5 ms, 5–10 psi) were then delivered every minute at different holding potentials (–80 to +20 mV, 20 mV increment). A linear regression of I_Gly_ amplitude plotted against the holding potential was used to analyse the voltage dependence. The intercept of the I–V relation with the abscissa was then extrapolated as *E*_Cl_. (As experiments were performed in bicarbonate-free solution buffered with HEPES I_Gly_ was solely mediated by Cl^−^ and thus we report *E*_Cl_). E_Cl_ predicted from the Nernst equation was −21.3 mV. Data are expressed as mean±s.e.m and were considered significant if *P*<0.05.

### Immunoblotting studies

The full-length human wild-type KCC2 and the three mutant KCC2 sequences were cloned into the mammalian expression vector, pCMV–Myc. We amplified the sequences from the Origene KCC2 wild-type expression vector and the mutagenized vectors using the Platinum Taq DNA Polymerase High Fidelity (Life Technologies), custom-designed cloning primers (available on request) and 94 °C for 2 min followed by 30 cycles of 94 °C for 30 s, 55 °C for 30 s and 68 °C for 3 min 30 s. Then using the QIAquick Gel Extraction Kit (QIAGEN) purified PCR products and the pCMV–Myc plasmid were successively digested first with EcoRI and then with Acc65I (isoschizomer of KpnI) according to manufacturer's protocol. Following purification with the QIAquick Gel Extraction Kit the PCR products were ligated into the EcoRI and KpnI sites of the vector pCMV-Myc (which was kindly provided from Dr Paul Gissen) using T4 DNA ligase (Promega). For amplification of Myc-tagged KCC2 constructs we transformed NEB 10-beta competent E.coli cells (New England BioLabs) with the plasmids and subsequently isolated the plasmids with the QIAGEN Plasmid Maxi Kit (QIAGEN). All expression constructs were confirmed by Sanger sequencing of the entire coding region, to ensure that during PCR amplification no additional mutations had been introduced. LLC-PK cells (non-transfected LLC-PK1 cells kindly supplied by Dr Roxanne A. Vaughan, University of North Dakota) were then transiently transfected with equal amounts of WT, L311H, L426P and G551D constructs. After 24 h, surface proteins were labelled with biotin and cells were lysed. Biotinylated fraction and total cell lysates were eluted with 8% Tris-glycine, separated on a SDS polyacrylamide gel, transferred to nitrocellulose membrane and blotted with anti-myc antibodies. Control beta-actin bands were observed in the total cell lysate but not in the biotinylation assay (Fig. 4). Densitometric analysis with NIH Image J yielded Area-Under-Curve (AUC) for each western blot band. An uncropped immunoblot is included as Supplementary Figure 3. Two analyses were undertaken to determine the effect of mutations (Table 1):

1. Calculation of cell-surface KCC2 relative to the total level of KCC2 and normalization with WT values. In this analysis both bands are taken together for both surface and total expression, assuming full and equal recovery through biotinylation steps in work-up 
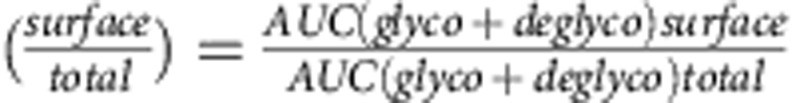
2. Calculation of the fraction of glycosylated state in the cell-surface (1), or total expression, (2):

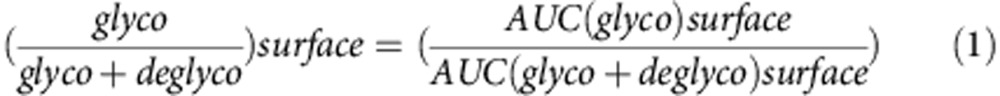



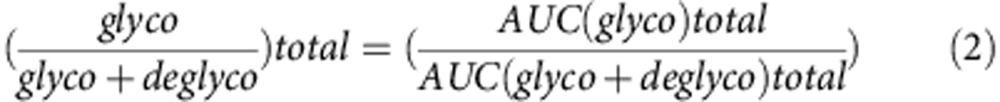


### Immunostaining and confocal microscopy

HEK293 cells were transfected with pCAGGS-KCC2-FLAG^33^ (gift from S. Levi, Institut National de la Santé et de la Recherche Médicale, Paris, France), pCAGGS-KCC2-FLAG-L311H, pCAGGS-KCC2-FLAG L426P or pCAGGS-KCC2-FLAG G551D and HcRed NLS using FuGENE (Promega). After 24 h, cells were fixed in 4% (w/v) paraformaldehyde in phosphate buffered saline (PBS) for 15 s, washed with PBS, incubated with 50 mM NH_4_Cl for 10 min, washed with PBS and blocked for 30 min in PBS supplemented with 2% (w/v) bovine serum albumin (BSA) and 10% (v/v) goat serum. Total KCC2-FLAG in cells was assessed by the addition of 1% (v/v) Triton X-100 to blocking media. Cells were incubated for 1 h with rabbit anti-FLAG antibody (1:200; Sigma, catalogue number F7425) in PBS with 2% BSA, washed in PBS, incubated with AlexaFluor 488-conjugated goat anti-rabbit antibody (1:600; Invitrogen, catalogue number A-11008) in PBS-BSA, washed with PBS and mounted on slides. Images were collected using a Zeiss LSM 710 confocal microscope using × 63 objective.

### Zebrafish models and behavioural analysis

Zebrafish (*Danio rerio*, AB strain) obtained from the Zebrafish International Resource Center (Eugene, OR) were maintained at 28.5 °C following established procedures. The experiments were performed in accordance with the approved guidelines of the Committee on Animal Use and Care at the National Institute of Genetics, Japan. Zebrafish have two KCC2 orthologs (KCC2a and KCC2b), due to an ancient gene duplication. TALENs for zebrafish KCC2a and KCC2b were designed to target the fourth exon of both *KCC2a* and *KCC2b* genes. To construct TALEN plasmid, TAL effector repeats were assembled in pFUS plasmid by BsaI (New England Labs) digestion and following T4 DNA ligase reaction were subsequently subcloned into pCS2+ vector using BsmBI (New England Labs) restriction sites^34^. The TALEN constructs were linearized by NotI digestion and used for in vitro transcription. Capped RNAs were synthesized using mMESSAGEmMACHINE SP6 kit (Life Technologies) according to the manufacturer's instruction. TALEN RNAs (200 pg for each TALEN) were injected into 1-cell stage zebrafish embryos and gene disruption of KCC2a (8 bp deletion) and KCC2b (five base deletion) (Supplementary Data 1) was confirmed by sequencing the F1 fish. KCC2a-KCC2b double knockout larvae were obtained by crossing double heterozygous carrier fish. Genotyping was done using following primers and PCR products were separated by 15∼20% polyacrylamide gel electrophoresis.

KCC2a (genotyping, forward): 5′-TGGGTGTGTACCTGCCC-3′ KCC2a (genotyping, reverse): 5′-CCTGAGGAATAGAATCACACCC-3′ KCC2b (genotyping, forward): 5′-CGTTTACCTACCATGTCTTCAAAACAT-3′ KCC2b (genotyping, reverse): 5′-CCAGGTCATCCTGAGGAACAAA-3′ At 2 days post fertilization, the embryonic behaviour of the wild-type and KCC2a-KCC2b double knockout embryos (both male and female, 10 per group) were investigated by a tactile response assay in which mechanosensory stimulation was applied to the tail. The response was recorded using a dissection microscope (MZ16, Leica) and a high-speed CCD camera (HAS-220, Ditect).

## Additional information

**How to cite this article:** Stödberg, T. *et al*. Mutations in *SLC12A5* in epilepsy of infancy with migrating focal seizures. *Nat. Commun.* 6:8038 doi: 10.1038/ncomms9038 (2015).

## Supplementary Material

Supplementary Figures, Tables, Notes, Methods and References Supplementary Figures 1-3, Supplementary Tables 1-4, Supplementary Note 1, Supplementary Methods and Supplementary References

Supplementary Data 1 cDNA and protein sequences for zebrafish Kcc2a and Kcc2b (wild-type and knockout)

Supplementary Movie 1 Wild-type zebrafish larvae showed typical escape swimming upon touch with rhythmic side-to-side contraction of the trunk.

Supplementary Movie 2 KCC2a-KCC2b double knockout zebrafish showed abnormal jerky spasmodic movements during the escape response at 2dpf.

## Figures and Tables

**Table 1:** Quantification of total and glycosylated sub-fraction of KCC2 at the cell surface and in whole cell lysate.

	**Glycosylated plus unglycosylated KCC2 at the surface (fraction of total, % of wild-type)**	**Glycosylated KCC2 (% of glycoslyated plus unglycosylated)**	
		**Surface fraction**	**Total fraction**
WT	100^±^±0%	50±13%	43±1%
L311H	34±7%**	22±5%*	17±1%**
G551D	41±10%*	32±7%	38±1%*
L426P	55±13%*	10±3%**	16±4%*

WT, wild-type.

*n*=4. *P<0.05, **P<0.005 (compared with WT, one-sample Student's *t*-test).

All three mutants showed significantly reduced levels of cell-surface over total expression of KCC2 when compared with WT with reduced levels of glycosylated KCC2 protein evident at the cell surface and also in total cell lysates (Figure 4).
