# Supplementary material for: Mutations in SLC12A5 in epilepsy of infancy with migrating focal seizures
Source: Nat Commun. 2015 Sep 3;6:8038. doi: 10.1038/ncomms9038 (PMC4569694; doi:10.1038/ncomms9038)
Supplement: Supplementary Figures, Tables, Notes, Methods and References — Supplementary Figures 1-3, Supplementary Tables 1-4, Supplementary Note 1, Supplementary Methods and Supplementary References [file ncomms9038-s1.pdf]

Supplementary Information

Supplementary Figures

1a

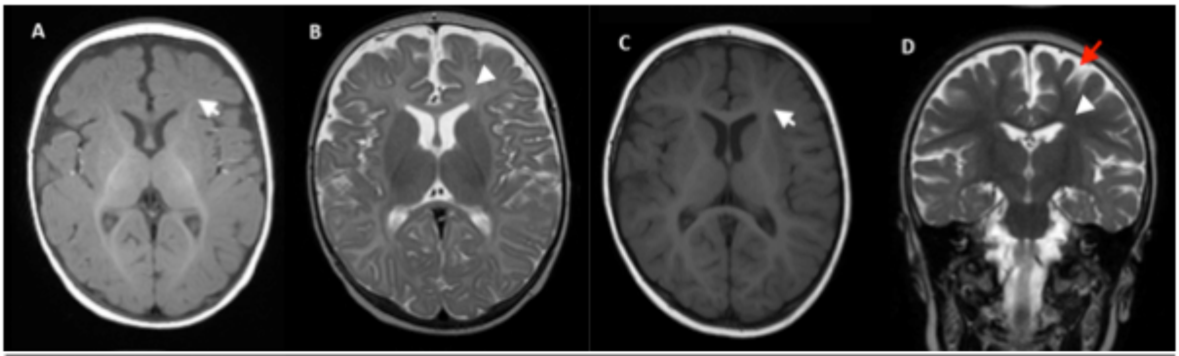

1b

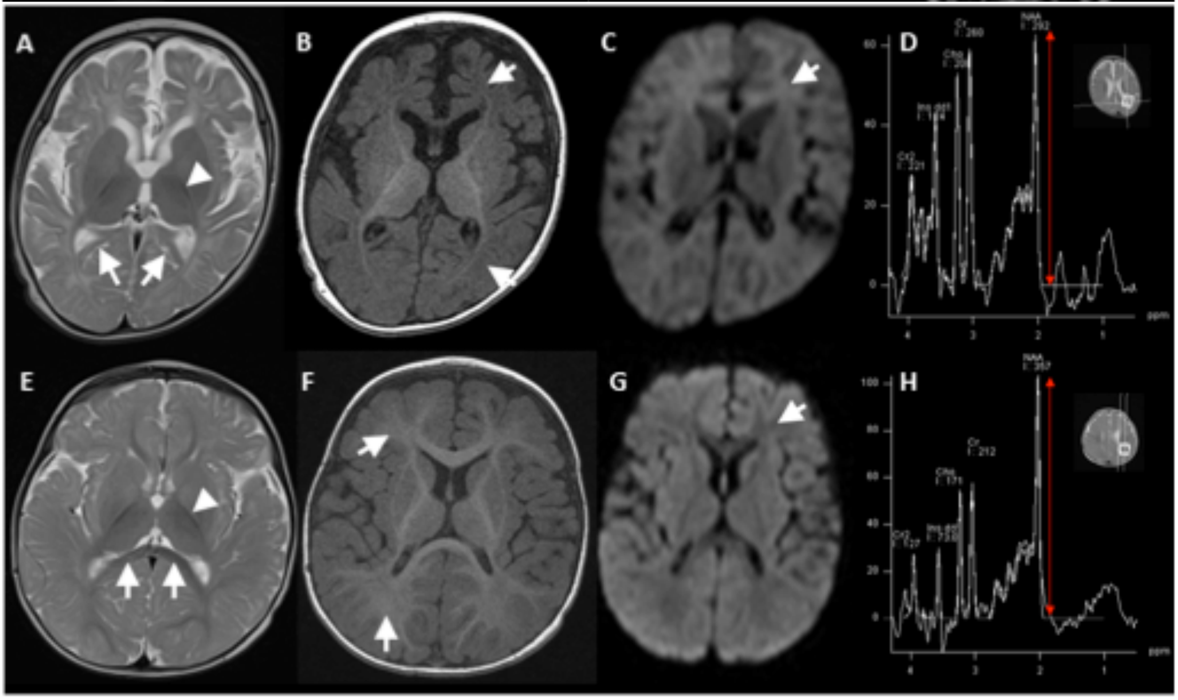

Supplementary Figure 1:

MR imaging and spectroscopy in *KCC2*-MPSI reveal global cerebral atrophy and delayed myelination

1a: Selected images from Patient A-II:1 at 4 months (A&B) and 29 months (C&D). The axial T1 and T2 images show delayed myelin maturation in the white matter (A arrow & B arrowhead). By 29 months (C arrow and D arrowhead), myelination has progressed. However the coronal T2 (D) shows that despite myelin maturation

there remains significant prominence of the extra-axial CSF space due to a reduction in overall brain volume (red arrow) in keeping with global cerebral atrophy.

1b: Selected images from Patient B-II:4 at 8 months (A-D) and a normal age-matched control (E-H). The axial T2 sequences (A&E) demonstrate reduced myelination in the posterior limb of the internal capsule (arrowhead) compared to the normal with lack of myelination in the posterior fornices heading towards the splenium of the corpus callosum (arrows). The axial T1 (B&F) further show this delayed myelination in the frontal and occipital white matter (arrows) as reduced signal in the patient compared to normal. The diffusion imaging (C&G) demonstrates increased signal in the white matter compared to the cortex (arrows) in the patient compared to the control subject. Spectroscopy in the occipital white matter (D&H) demonstrates a relatively normal choline (Cho) /creatine (Cr) ratio, but marked reduction in the relative N-acetyl aspartate peak (NAA-red arrow). These findings are consistent with delayed myelin maturation throughout the developing brain in the affected patient.

L311

272	VKYVNKFALVFLGCVILSILAIYAGVIKSAFDPPNFPICLLGNRTLSRHG	321	SLC12A5, H. SAPIENS
249	VKYVNKFALVFLGCVILSILAIYAGVIKSAFDPPNFPICLLGNRTLSRHG	298	SLC12A5, P. TROGLODYTES
302	VKYVNKFALVFLGCVILSILAIYAGVIKSAFDPPNFPICLLGNRTLSRHG	351	SLC12A5, C. LUPUS
242	VKYVNKFALVFLGCVILSILAIYAGVIKSAFDPPNFPICLLGNRTLSRHG	291	SLC12A5, B. TAURUS
249	VKYVNKFALVFLGCVILSILAIYAGVIKSAFDPPNFPICLLGNRTLSRHG	298	SLC12A5, M. MUSCULUS
272	VKYVNKFALVFLGCVILSILAIYAGVIKSAFDPPNFPICLLGNRTLSRHG	321	SLC12A5, R. NORVEGICUS
268	VKYVNKFALVFLGCVILSILAIYAGVIKSAFDPPSFPICLLGNRTLSRHG	317	SLC12A5, G. GALLUS
236	VKYVNKLALVFLACVILSILAIYAGVIKTSFDPPDFPVCVLGNRTLVSKA	285	LOC797331, D. RERIO
207	VKYVNKLALVFLACVICSILAVYAGVIKTAFEPPVFPVCVLGNRTLVWKG	256	LOC572215, D. RERIO

L426

422	YFTLLVGIYFPSVTGIMAGSNRSGDLRDAQKSIPTGTILAIATTSAVYIS	471	SLC12A5, H. SAPIENS
399	YFTLLVGIYFPSVTGIMAGSNRSGDLRDAQKSIPTGTILAIATTSAVYIS	448	SLC12A5, P. TROGLODYTES
452	YFTLLVGIYFPSVTGIMAGSNRSGDLRDAQKSIPTGTILAIATTSAVYIS	501	SLC12A5, C. LUPUS
392	YFTLLVGIYFPSVTGIMAGSNRSGDLRDAQKSIPTGTILAIATTSAVYIS	441	SLC12A5, B. TAURUS
399	YFTLLVGIYFPSVTGIMAGSNRSGDLRDAQKSIPTGTILAIATTSAVYIS	448	SLC12A5, M. MUSCULUS
422	YFTLLVGIYFPSVTGIMAGSNRSGDLRDAQKSIPTGTILAIATTSAVYIS	471	SLC12A5, R. NORVEGICUS
417	YFTLLVGIYFPSVTGIMAGSNRSGDLRDAQKSIPTGTILAIATTSAVYIS	466	SLC12A5, G. GALLUS
385	FFTLLVGIYFPSVTGIMAGSNRSGDLQDAQKSIPVGTILAITTTSIIYMS	434	LOC797331, D. RERIO
356	FFTMLVGIYFPSVTGIMAGSNRSGDLRDAQKSIPIGTILAITTTSIIYMS	405	LOC572215, D. RERIO

G551

522	GLQSLTGAPRLLQAISRDGIVPFLQVFGHGKANGEPTWALLLTACICEIG	571	SLC12A5, H. SAPIENS
499	GLQSLTGAPRLLQAISRDGIVPFLQVFGHGKANGEPTWALLLTACICEIG	548	SLC12A5, P. TROGLODYTES
552	GLQSLTGAPRLLQAISRDGIVPFLQVFGHGKANGEPTWALLLTACICEIG	601	SLC12A5, C. LUPUS
492	GLQSLTGAPRLLQAISRDGIVPFLQVFGHGKANGEPTWALLLTACICEIG	541	SLC12A5, B. TAURUS
499	GLQSLTGAPRLLQAISRDGIVPFLQVFGHGKANGEPTWALLLTACICEIG	548	SLC12A5, M. MUSCULUS
522	GLQSLTGAPRLLQAISRDGIVPFLQVFGHGKANGEPTWALLLTACICEIG	571	SLC12A5, R. NORVEGICUS
517	GLQSLTGAPRLLQAISRDGIVPFLRVFGHGKANGEPTWALLLTACICEIG	566	SLC12A5, G. GALLUS
485	GLQSLTGAPRLLQAIARDGIIPFLRVFGHGKANGEPTWALLLTACICESG	534	LOC572215, D. RERIO
456	GLQSLTGAPRLMQAIARDGIVPFLRVFGHGKANGEPTWALLLTACICESG	505	LOC572215, D. RERIO

Supplementary Figure 2:

Conservation in species for identified mutations

The mutated amino acid residues L311, L426 and G551 are highly conserved in *SLC12A5* between species. ENSEMBL-id for the different species are: *H. sapiens*, ENSP00000387694; *P. troglydytes*, ENSPTRP00000023337; *C. lupus*, ENSCAFP00000014673; *B. taurus*, ENSBTAP00000018852; *M. musculus*, ENSMUSP00000096690; *R. norvegicus*, ENSRNOP00000062103; *G. gallus*, ENSGALP00000011213; *D. rerio* (loc797331), ENSDARP00000119636; *D. rerio* (loc572215), ENSDARP00000057257.

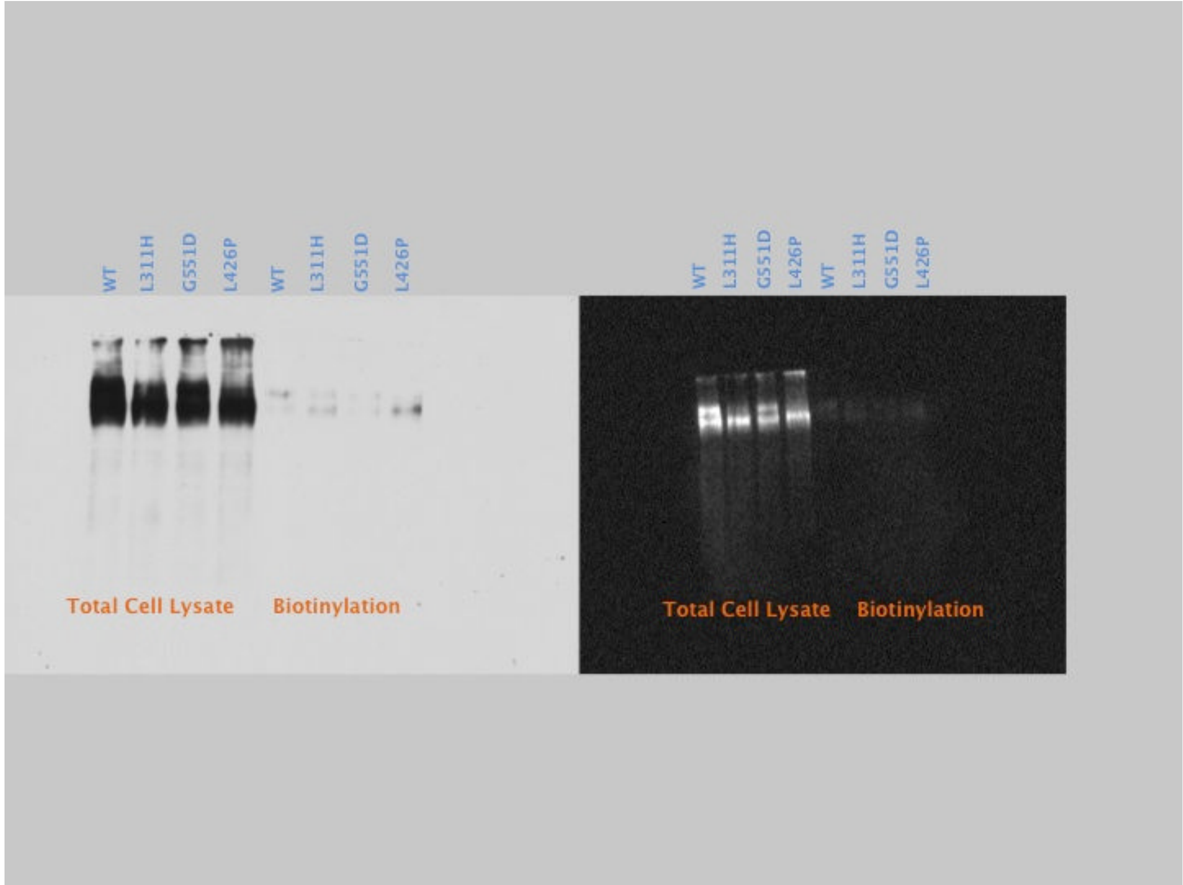

Supplementary Figure 3:

Uncropped immunoblot for cell surface biotinylation and total cell lysate studies (cropped version depicted in Figure 4)

Supplementary Tables

Supplementary Table 1: Genes reported in Migrating Partial Seizures of Infancy (MPSI)

Gene<br>OMIM<br>EIEE OMIM number	Number of reported cases	Mode of inheritance	Effect on protein function	Protein	Protein function	Reference
KCNT1<br>MIM 608167<br>EIEE 14	10	Autosomal dominant, usually *de novo*	Gain-of-function	Sodium activated potassium channel (K_{Na})	Outwardly rectifying potassium channel subunit, required for hyperpolarisation following repetitive firing<br>Interaction with wider protein network including FMRP	1,2,3
SCN1A<br>MIM 182389	3	Autosomal dominant, usually *de novo*	Likely to be loss-of-function	Type 1 voltage-gated sodium channel alpha subunit	Initiation and propagation of neuronal action potentials, particularly in GABAergic interneurons	4,5
TBC1D24<br>MIM 613577<br>EIEE 16	2	Autosomal recessive	Loss-of-function	TBC domain containing RAB-specific GTPAse activating protein	Regulation of neurite growth and branching, morphological and functional maturation of neuronal circuits	6
SLC25A22<br>MIM 609302<br>EIEE 3	2	Autosomal recessive	Loss-of-function	Mitochondrial glutamate transporter	Catalyses the co-transport of glutamate with H^+ or exchanges glutamate for OH^-	7
SCN2A<br>MIM 182390<br>EIEE 11	1	Autosomal dominant, usually *de novo*	Likely to be gain-of-function	Type 2 voltage-gated sodium channel alpha subunit	Initiation and propagation of neuronal action potentials, located at axon initial segment	8
PLCB1<br>MIM 607120<br>EIEE 12	1	Autosomal recessive	Loss-of-function	Phospholipase C-beta type 1	Catalyzes the generation of inositol 1,4,5-trisphosphate (IP3) and diacylglycerol (DAG) from phosphatidylinositol 4,5-bisphosphate (IP2), important in the intracellular transduction of many extracellular signals	9
SCN8A<br>MIM 600702<br>EIEE 13	1	Autosomal dominant, *de novo*	Likely to be gain-of-function	Type 8 voltage-gated sodium channel alpha subunit	Initiation and propagation of neuronal action potentials, particularly in GABAergic interneurons; expressed later in post-natal development than *SCN2A*	10
QARS<br>MIM 603727<br>MIM 615760	2	Autosomal recessive	Loss-of-function	Amino-acyl tRNA synthetase	Ensure translational fidelity by matching of tRNA with correct amino acids<br>Loss of function leads to impaired neuronal survival	11

Supplementary Table 2:

Annotations and score parameters used by MIP in exome data analysis

**Annotation**	**Rank score parameter**	**Source**
Ensemble gene ID	No	Ensemble
HGNC gene symbol	No	HGNC[1]
HGNC gene name	No	HGNC[1]
HGNC gene name synonyms	No	HGNC[1]
OMIM gene description	No	OMIM[2]
OMIM morbid description	No	OMIM[2]
HGMD accession	Yes	HGMD[3]
HGMD variant type	No	HGMD[3]
HGMD variant associated pubmed ID	No	HGMD[3]
Gene annotation	Yes	ANNOVAR
Functional annotation	Yes	ANNOVAR
Transcript and protein annotation	No	ANNOVAR
Phast cons elements	Yes	ANNOVAR
GERP[4] elements	Yes	ANNOVAR
Segmental duplications	Yes	ANNOVAR
1000Genomes MAF[5]	Yes	ANNOVAR
DbSNP MAF[5]	Yes	ANNOVAR
DbSNP nonflagged	Yes	ANNOVAR
Esp6500[6] MAF[5]	Yes	ANNOVAR
SIFT	Yes	ANNOVAR
PolyPhen[7]	Yes	ANNOVAR
MutationTaster	Yes	ANNOVAR
GERP[4]	Yes	ANNOVAR
LRT[8]	Yes	ANNOVAR
PhyloP[9]	Yes	ANNOVAR
Transfac[10]	No	ANNOVAR
snoRNA & miRNA annotations	No	ANNOVAR
GT[11] call filter	Yes	GATK
GT[11] Call	Yes	GATK
Genetic inheritance models	Yes	MIP
Disease group	No	dbCMMS
Clinical db genome build	No	dbCMMS
Disease gene model	No	dbCMMS
Clinical db gene annotation	No	dbCMMS

[1]HGNC: HUGO gene nomenclature committee, [2]OMIM: Online mendelian inheritance in man, [3]HGMD: Human gene mutation database, [4]GERP: Genomic Evolutionary Rate Profiling, [5]MAF: Minor allele frequency, [6]ESP: NHLBI GO Exome Sequencing Project, [7]PolyPhen: Polymorphism Phenotyping, [8]LRT: likelihood ratio test, [9]PhyloP: phylogenetic p-values, [10]Transfac: Transcription Factor Binding Sites, [11]GT: Genotype

Supplementary Table 3a: Number of variants in analysis for Family A

Stage	No. of variants	Criteria
**1**	**1639**	**Variant call quality[1], coverage[2], genetic model[3]**
**2**	**277**	**Passing stage 1, exonic and nonsynonymous**
**3**	**10**	**Passing stage 1,2, MAF[4] < 0.01 and SIFT < 0.05**
**4**	**2**	**Passing stage 1,2,3 and valid recessive pair[5]**

[1]Variant call quality: Passing the variant call quality threshold in the variant calling
[2]Coverage: 10x coverage for assessed allele
[3]Genetic model: Correct segregation according to classical Mendelian inheritance patterns (Supplementary: Gene models)
[4]MAF: Minor allele frequency in 1000G phase 1 and local frequency database
[5]Valid recessive pair: Each variant in the recessive pair must meet the criteria from stage 1-3

Supplementary Table 3b: Filtering of whole exome data for Family B

Chromosome	Start of homozygous region	End of homozygous region	Size of homozygous region (Mb)	Candidate genes from whole exome (homozygous model) before filtering using dbSNP	Candidate genes from whole exome (homozygous model) after filtering with dbSNP	Nature of Candidate Gene	Nature of mutation in candidate gene	Polyphen2, SIFT Provean2, Mutation Taster scores (if applicable)
3	156,581,034	172,339,752	16	None	None	-	-	-
4	48,283	10,728,759	10	*ZNF732, WFS1, CPZ, LOC650923*	*CPZ*	Carboxypeptidase Widely expressed in tissues Role in proteolysis and Wnt signalling pathway	Missense	PolyPhen2 = 0 (benign) SIFT= 0.09 (tolerated) Mutation Taster = 1.05 (N- denotes polymorphism)
6	13,361,927	56,208,751	43	*AK309533, HLA-A, HLA-B,MICA, TNXB, HLA-DRB5, HLA-DRB1, HLA-DQA1, HLA-DQB2, HLA-DPA1, HLA-DPB1, SLC26A8, KIAA0240*	*SLC26A8*	Testis anion transporter- implicated in spermatogenic failure	Missense	PolyPhen2 = 0.071 (benign) SIFT= 0.81 (tolerated) Mutation Taster =3.06 (N- denotes polymorphism)
7	146,711,789	152,600,817	6	None	None	-	-	-
16	49,866,005	65,809,044	15	None	None	-	-	-
**20**	**40,900,992**	**47,171,338**	**7**	***SLC12A5***	***SLC12A5***	**Neuronal-specific potassium-chloride co-transporter**	**Missense**	**PolyPhen2 = 0.994 (probably damaging) SIFT = 0.0 (deleterious) Provean = -4.420 (damaging)**
21	10,734,842	26,514,597	15	*POTED*	*POTED*	Ankyrin repeat domain-containing protein 21, prostate-, ovary-, testis-, and placenta-expressed gene	4 Missense changes in exon 1	PolyPhen2 = 0.04, 0.001, 0, 0.022 (all benign) SIFT = 0, 0, 0.66 (all tolerated), 0.98 (deleterious) Mutation Taster = 0.001146, 1, 2.2, 3.8 (N- denotes polymorphism)

Supplementary Table 4a: Cloning primers

SLC12A5-EcoRI	5'-ggcgaattcgccacc**atg**ctaaacaacctgacggac-3’
SLC12A5-SalI	5'-tccgtcgac**tca**ggagtagatggtgatgac-3'

Supplementary Table 4b: Mutagenesis primers

**Mutation**	**Primer Sequence**
SLC12A5-pLeu311His-F	ttcccgatctgccacctgggtaaccgc
SLC12A5-pLeu311His-R	gcggttacccaggtggcagatcgggaa
SLC12A5-pLeu426Pro-F	tcctacttcaccctgccggttggcatctacttc
SLC12A5-pLeu426Pro-R	gaagtagatgccaaccggcagggtgaagtagga
SLC12A5-pGly551Asp-F	caggtctttggccatgacaaggccaatggagag
SLC12A5-pGly551Asp-R	ctctccattggccttgtcatggccaaagacctg

Supplementary Note 1

Clinical description of MPSI cases

In Family A, two children with MPSI were born to non-consanguineous Caucasian parents. The elder child (A-II:1), a 9.6 year old boy, was the result of a normal term pregnancy and neonatal period; early psychomotor development was normal. At 3 months, the first seizure occurred during a febrile illness and consisted of brief behavioral arrest, unresponsiveness, and eye deviation. Subsequently, the child experienced afebrile hemiclonic seizures. Within 3 weeks, focal seizures became increasingly frequent and arose from different locations in both hemispheres. The semiology included behavioral arrest, hemiclonic twitching, tonic and atonic features, and eye deviation. Lateralizing signs sometimes switched side during ongoing seizures. The patient was hospitalized for 5 months with several periods of recurrent pharmacoresistant status epilepticus. Regression of motor and cognitive skills was observed.

Two EEGs at presentation were normal. Subsequent EEGs revealed status epilepticus with migrating foci, both with and without clinical correlate. Seizure activity sometimes showed intraictal migration between hemispheres (**Figure 1b**). Interictal background showed diffuse slowing and multifocal spikes. MRI brain at 4 months showed delayed myelination and at 29 months, global cerebral atrophy (**Supplementary Figure 1a**).

Multiple anti-epileptic medications were ineffective. At 8 months, intravenous methylprednisolone (30 mg per kg IV daily for 3 consecutive days) followed by oral prednisolone produced a marked reduction in seizure frequency. An EEG one

month after commencement of steroids showed no epileptiform activity. Over the next few years, 2.5 mg oral prednisolone on alternate days was maintained. Seizure frequency ranged from 1-79 per month, occurring in clusters over 1-7 days/month with seizure-free periods in between. The ketogenic diet, introduced at 5 years, reduced seizures to one per night between midnight and 7 am. He was more alert and interactive, and prednisolone was withdrawn. He is currently treated with sodium valproate and clobazam. Tapering of the ketogenic diet has been associated with a moderate rise in seizure frequency.

Patient A-II:1 made slow developmental gains. He crawled at 1.8 years of age, and walked independently from the age of 2.8 years. He was able to grasp for objects. He had no dysmorphic features apart from acquired microcephaly (birth head circumference 2.5th percentile, current head circumference 0.1st percentile). He was hypotonic with normal tendon reflexes. He smiled responsively and made eye contact but was non-verbal.

The younger sister, Patient A-II:2, currently aged 7.9 years, presented with a similar MPSI phenotype. At 4 months, she developed focal seizures that rapidly worsened and she was hospitalized for 3 months due to frequent drug-resistant migrating focal seizures. Steroids, including IV methylprednisolone, oral prednisolone and tetracosactide showed no benefit. At 7 months, she was on vigabatrin, zonisamide and clonazepam. Seizures clustered and she also responded to the ketogenic diet. Her motor development is more impaired as she is non-ambulatory nor does she grasp objects.

In Family B, the proband (B-II:4) was born following a normal pregnancy and delivery. He is the second child of first cousin Pakistani parents, and had an affected older brother (whose history and clinical presentation was re-examined in light of his younger brother's similar presentation) and two unaffected brothers (**Figure 1a**). He presented at 10 weeks with migrating focal seizures characterized by facial and limb twitching associated with excessive salivation. These were brief, lasting approximately 20 seconds, and frequent, occurring up to 70 times per day. His developmental progress prior to seizure onset was satisfactory but following seizure onset, regression occurred. Examination revealed a head circumference of 38 cm (0.4th percentile) with weight on the 10th percentile, global hypotonia, and normal antigravity movements.

EEG performed at 4 weeks revealed a slow background with multifocal spikes. Ictal EEG revealed consecutive seizures with onset in the central/vertex region followed by left hemisphere onset associated with facial twitching (**Figure 1b**). MRI of the brain at eight months showed significantly delayed myelination and increased white matter signal (**Supplementary Figure 1b**). Magnetic resonance spectroscopy (MRS) revealed a relatively reduced N-acetyl aspartate (NAA) peak (**Supplementary Figure 1b**).

Phenobarbitone was commenced initially with some reduction in seizure frequency but this was not sustained. Trials of pyridoxine and folinic acid were ineffective, as were levetiracetam and sodium valproate. The addition of topiramate to phenobarbitone led to a period of relative stability, but clusters of focal seizures continued particularly during febrile illnesses. Currently, he has brief focal seizures

consisting of twitching of the eyelids, face or either arm, which become more prolonged and intrusive and evolve to bilateral convulsive seizures during intercurrent illnesses.

At 17 months, he is significantly developmentally delayed but can fixate visually. There is evidence of ongoing regression with the gradual loss of swallowing skills over recent months. Examination reveals pyramidal signs on the left with microcephaly (head circumference 42 cm at 14 months, below 0.4th percentile).

The proband's oldest brother (B-II:1) presented similarly at 6 weeks. Following early normal development, he had frequent, migrating focal seizures of the face and limbs occurring in clusters with intermittent evolution to bilateral convulsions. Twitching of the tongue, apnea and drooling were prominent ictal features. Seizures were refractory to multiple antiepileptic drugs and there was no response to pyridoxine. An EEG at 10 weeks captured two seizures with eye deviation to the right, chewing automatisms, and right eyelid twitching followed by unresponsiveness with loss of tone. Epileptiform activity over the left temporal region was captured, and other EEGs captured similar seizures with ictal activity in other foci. CT brain at 10 weeks showed progressive generalized atrophy compared to CT at 6 weeks. MRI brain at 10 weeks revealed prominent extra-axial CSF spaces with evidence of thin subdural collections (hygromas) bilaterally, the right slightly larger than the left. There were no structural abnormalities. Head circumference at birth was 36 cm (50th percentile) but by 6 months was 40.5 cm (below the 0.4th percentile). He died at 2.5 years following a chest infection, increased seizures and cardiac arrest.

Supplementary Methods

Exome sequencing was undertaken for the parents and the affected siblings in Family A and bioinformatic analysis was performed using the Mutation Identification Pipeline v.1.5.6 http://mip-api.readthedocs.org/en/latest/index.html. Approximately 50 gigabases (Gb) of sequencing data was produced, generating an average 90-190 fold coverage of the exome within the family. The exome was sufficiently covered at 95.8% of the bases according to our cut-off limits (10x read depth) and 135,851 variants were called. 86% (116,691 variants) were single nucleotide variants (SNV) and 14% (19,160 variants) were indels. All variants were scored and ranked using MIPs weighted sum model (**Supplementary Table 2**), which uses multiple parameters, but emphasizes Mendelian inheritance patterns, conserved, rare and protein damaging variants. This left only one gene, *SLC12A5,* with adequate coverage in all individuals sequenced that conformed to autosomal recessive inheritance. *SLC12A5* also stood out due to its predicted critical function in neuronal chloride homeostasis and relationship to GABAergic and glycinergic transmission. The mutations found were c.1277T>C resulting in the amino acid change L426P, and c.1652G>A change, causing the G551D substitution. As confirmed by Sanger sequencing, both affected siblings in Family A were compound heterozygous for both mutations (**Figure 1c,d**). L426P was inherited maternally and the G551D paternally. Neither mutation was present in 200 ethnically matched control chromosomes. In addition, these variants are not reported in established variant databases (including dbSNP137, 1000 Genomes SNP calls and the NHLBI Exome Sequencing Project) and are both highly conserved (**Supplementary Figure 2**). L426P is predicted to be damaging (score 1.000, sensitivity: 0.00; specificity: 1.00) using PolyPhen2

(http://genetics.bwh.harvard.edu/pph2). PROVEAN score was -6.554 (http://provean.jcvi.org/index.php) and variants with a score equal to or below -2.5 are considered "deleterious”. SIFT score (http://sift.bii.a-star.edu.sg) was 0 (probabilities less than 0.05 predicted to be deleterious). G551D was also predicted to be damaging, with a PolyPhen2 score of 0.931 (sensitivity: 0.81; specificity: 0.94) and PROVEAN score -4.020. In addition, no pathogenic variants in genes causing MPSI (**Supplementary Table 1**) and other early infantile epileptic encephalopathies were identified on exome sequencing analysis.

SNP array analysis in Family B revealed 7 regions of common homozygosity (**Supplementary Table 3b**). These regions were not shared in the unaffected family members. Exome sequencing was performed in Patient B-II:4 with an average 32.7-fold coverage of the exome; 2x coverage in 93.5% of bases and 10x coverage in 84.0% of bases according to our cut-off limits described in the Methods Section. Further analysis of the filtered variants was undertaken to determine which occurred within the 7 linked homozygous regions. 21,381 exonic or splice site variants were identified, of which 1173 were non-synonymous or stop-gain. Of these, 262 variants fitted a homozygous model. Analysis for changes within the homozygous regions identified revealed 18 potential candidate genes. After filtering against dbSNP135 for synonymous changes, 4 candidate genes remained (*SLC12A5*, *CPZ, SLC26A8, POTED*) and *SLC12A5* was determined to be by far the most biologically plausible for our clinical phenotype of infantile onset epilepsy (**Supplementary Table 3b**). The single homozygous variant identified in *SLC12A5,* c.932T>A, results in the missense change L311H (**Figure 1c**) occurring within a 7Mb homozygous region on chromosome 20. Appropriate familial

segregation was verified using Sanger sequencing. This variant is not reported in established variant databases (including dbSNP137, 1000 Genomes SNP calls and the NHLBI Exome Sequencing Project) and is highly conserved (**Supplementary Figure 2**). Further analysis indicated that L311H is predicted to be damaging with a PolyPhen2 score of 0.994 (sensitivity: 0.69; specificity: 0.97), SIFT score of 0.00 and Provean score of -4.420. As for Family A, no pathogenic variants in genes causing MPSI (**Supplementary Table 1**) and other early infantile epileptic encephalopathies were identified on exome sequencing analysis.

All 26 exons and exon/intron boundaries of *SLC12A5* were Sanger sequenced in 12 unrelated Swedish patients with EIEE (including 1 patient with MPSI) and 13 patients from the UK with MPSI. Exome sequencing data (undertaken at Duke University) for a further 24 patients from the UK, Australian and US MPSI cohorts were evaluated for putative pathogenic variants in *SLC12A5.* Overall, coverage was excellent for all *SLC12A5* coding exons, bar exons 1, 2, 16 and 17. Where possible, these exons were then sequenced by direct Sanger sequencing. No mutations were identified in these 24 patients, and variants R952H and R1049C were not identified in the screened cohort.

Supplementary References

1. Barcia, G. *et al*. De novo gain-of-function KCNT1 channel mutations cause malignant migrating partial seizures of infancy. *Nat. Genet*. **44**, 1255–1259 (2012).
2. McTague, A. *et al*. Migrating partial seizures of infancy: expansion of the electroclinical, radiological and pathological disease spectrum. *Brain* **136**, 1578–1591 (2013).
3. Ishii, A. *et al.* A recurrent KCNT1 mutation in two sporadic cases with malignant migrating partial seizures in infancy. *Gene* **531**, 467–471 (2013).
4. Carranza Rojo, D. *et al*. De novo SCN1A mutations in migrating partial seizures of infancy. *Neurology* **77**, 380–383 (2011).
5. Freilich, E.R. *et al.* Novel SCN1A mutation in a proband with malignant migrating partial seizures of infancy. *Arch. Neurol.* **68**, 665–671 (2011).
6. Milh, M. *et al*. Novel compound heterozygous mutations in TBC1D24 cause familial malignant migrating partial seizures of infancy. *Hum. Mutat.* **34**, 869–872 (2013).
7. Poduri, A. *et al*. SLC25A22 is a novel gene for migrating partial seizures in infancy. *Ann. Neurol.* **74**, 873–882 (2013).
8. Dhamija, R., Wirrell, E., Falcao, G., Kirmani, S. & Wong-Kisiel, L.C. Novel de novo SCN2A mutation in a child with migrating focal seizures of infancy. *Pediatr. Neurol.* **49**, 486–488 (2013).
9. Poduri, A. *et al.* Homozygous PLCB1 deletion associated with malignant migrating partial seizures in infancy. *Epilepsia* **53**, e146–150 (2012).
10. Ohba, C. *et al*. Early onset epileptic encephalopathy caused by de novo SCN8A mutations. *Epilepsia* **55**, 994-1000 (2014).

11. Zhang, X. *et al.* Mutations in QARS, encoding glutaminyl-tRNA synthetase, cause progressive microcephaly, cerebral-cerebellar atrophy, and intractable seizures. *Am. J. Hum. Genet.* ***94****,* 547-558 (2014).
